# Supplementary figures and images for: Continuous detection of Chikungunya Virus in a passive surveillance system in southern Thailand, 2012–2019
Source: PLoS Negl Trop Dis. 2025 Jan 7;19(1):e0012776. doi: 10.1371/journal.pntd.0012776 (PMC11741575; doi:10.1371/journal.pntd.0012776)

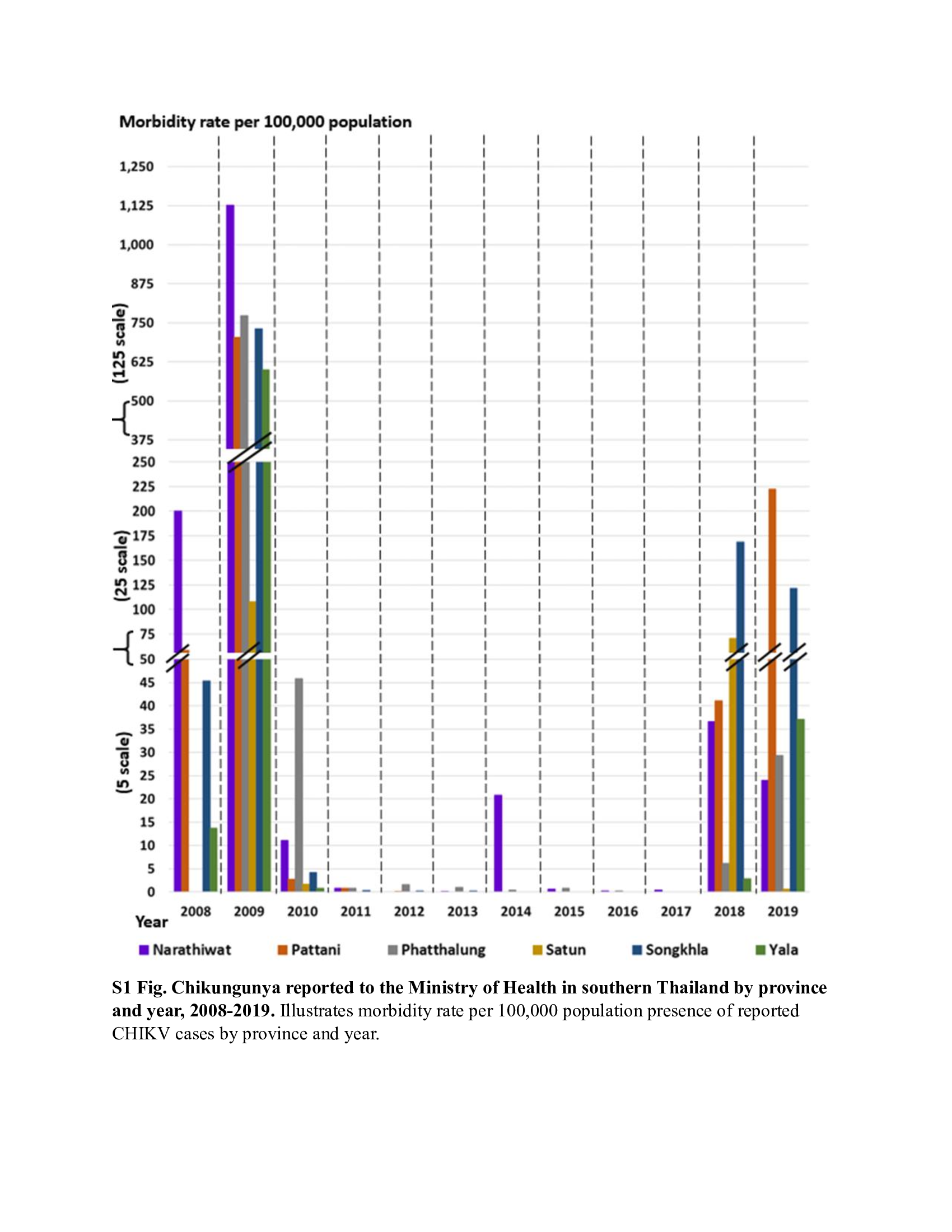

Supplement: S1 Fig — (TIFF) [file pntd.0012776.s001.tiff]

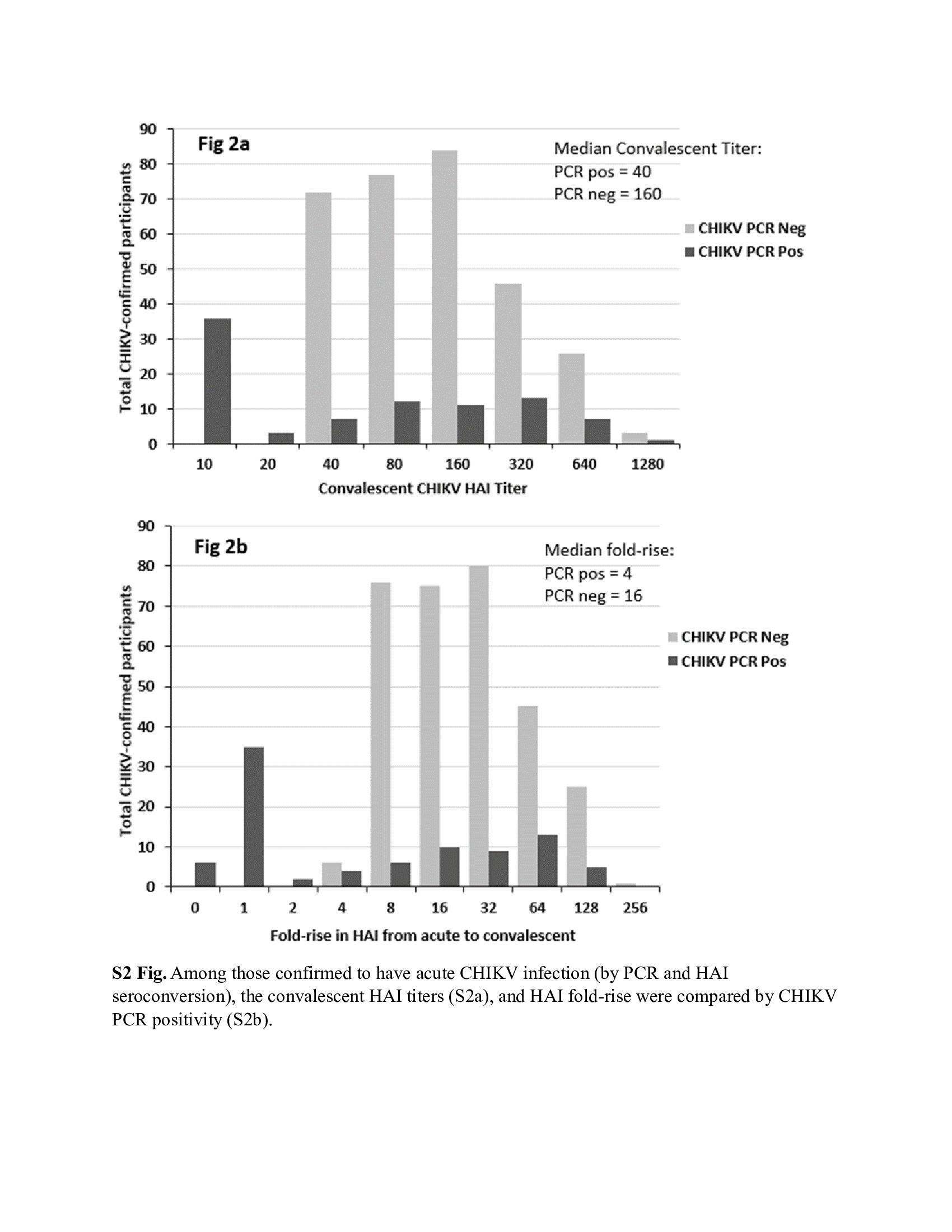

Supplement: S2 Fig — (TIFF) [file pntd.0012776.s002.tiff]

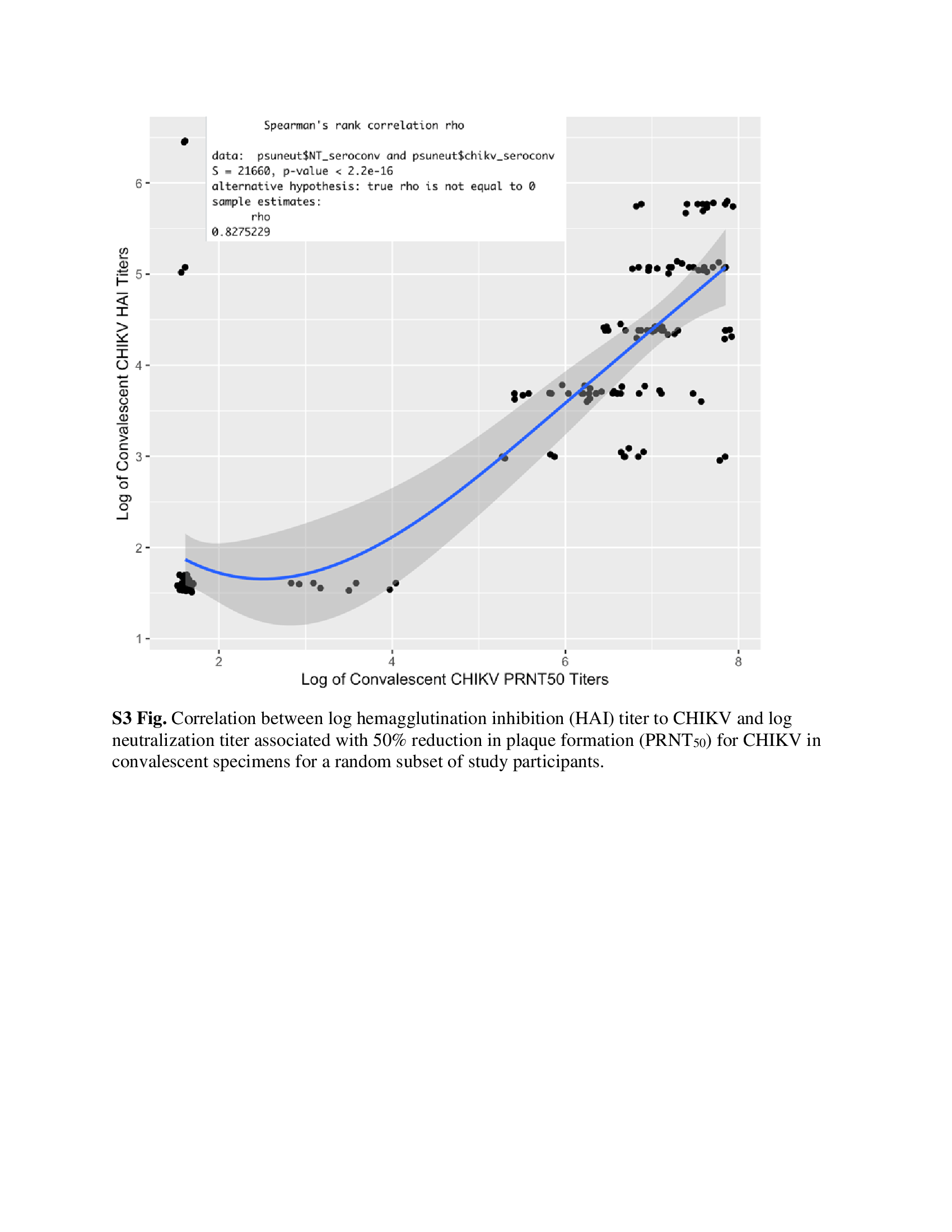

Supplement: S3 Fig — (TIFF) [file pntd.0012776.s003.tiff]
