# Supplementary material for: Continuous detection of Chikungunya Virus in a passive surveillance system in southern Thailand, 2012–2019
Source: PLoS Negl Trop Dis. 2025 Jan 7;19(1):e0012776. doi: 10.1371/journal.pntd.0012776 (PMC11741575; doi:10.1371/journal.pntd.0012776)
Supplement: Attachment — Submitted filename: Response to Technical Review 29 June 2024.docx [file pntd.0012776.s005.docx]

*Dear Dr Farmer,

Thank you for submitting your paper, "Continuous detection of Chikungunya Virus in a passive surveillance system in southern Thailand, 2012-2019", to PLOS Neglected Tropical Diseases. We have checked over your submission and, before we can assign your manuscript to the Editor, we ask that you first please make the following modifications:

1) Thank you for stating "Informed consent/assent was obtained per the primary protocol."

Please state "whether written/verbal consent was obtained
NOTE: If child participants, the statement must declare that formal consent was obtained from the parent/guardian.].*

The statement “Written informed consent/assent was obtained per the primary protocol, with parental/guardian consent and participant assent for children per local requirements” was added to line 130-131.

*2) We noticed that you used the phrase 'data not shown' in the manuscript. We do not allow these references, as the PLOS data access policy requires that all data be either published with the manuscript or made available in a publicly accessible database. Please amend the supplementary material to include the referenced data or remove the references.*

We have updated Lines 211-213 and Table 2 to show the results and will provide the data for publication via the respository. Also updated Lines 241-242 to reflect data can be found in the Supplemental Figure 1. *3) We do not publish any copyright or trademark symbols that usually accompany proprietary names, eg ©,  ®, or TM  (e.g. next to drug or reagent names). Therefore please remove all instances of trademark/copyright symbols throughout the text, including: SuperScript™ on line no 154, and IXCHIQ®. on line no 428.*

These symbols were removed. *4) Please upload all main figures as separate Figure files in .tif or .eps format. For more information about how to convert and format your figure files please see our guidelines:*
[*https://journals.plos.org/plosntds/s/figures*](https://journals.plos.org/plosntds/s/figures)

This has been completed and main figures uploaded as separate .tif files. *5) We have noticed that you have included [Figure 5].  However, there are no references to this figure in the manuscript PDF. Please ensure that all included figures are referenced to ensure that your paper is fully reviewed.*

This figure is referenced on line 273. “Twenty-one CHIKV viruses were successfully isolated and sequenced from participants in the study from 2012-2019; all belonged to the IOL of the ECSA strain (Fig 5).“
 *6) We have noticed that you have uploaded Supporting Information files, but you have not included a list of legends. Please add a full list of legends for your Supporting Information files after the references list.*

A list of legends has been provided after the reference list. *7) Some material included in your submission may be copyrighted. According to PLOSu2019s copyright policy, authors who use figures or other material (e.g., graphics, clipart, maps) from another author or copyright holder must demonstrate or obtain permission to publish this material under the Creative Commons Attribution 4.0 International (CC BY 4.0) License used by PLOS journals. Please closely review the details of PLOSu2019s copyright requirements here: PLOS Licenses and Copyright. If you need to request permissions from a copyright holder, you may use PLOS's Copyright Content Permission form.
Please respond directly to this email and provide any known details concerning your material's license terms and permissions required for reuse, even if you have not yet obtained copyright permissions or are unsure of your material's copyright compatibility. Once you have responded and addressed all other outstanding technical requirements, you may resubmit your manuscript within Editorial Manager.
Potential Copyright Issues:

i) Figure 1. Please (a) provide a direct link to the base layer of the map (i.e., the country or region border shape) and ensure this is also included in the figure legend; and (b) provide a link to the terms of use / license information for the base layer image or shapefile. We cannot publish proprietary or copyrighted maps (e.g. Google Maps, Mapquest) and the terms of use for your map base layer must be compatible with our CC BY 4.0 license.
Note: if you created the map in a software program like R or ArcGIS, please locate and indicate the source of the basemap shapefile onto which data has been plotted.
If your map was obtained from a copyrighted source please amend the figure so that the base map used is from an openly available source. Alternatively, please provide explicit written permission from the copyright holder granting you the right to publish the material under our CC BY 4.0 license.
If you are unsure whether you can use a map or not, please do reach out and we will be able to help you. The following websites are good examples of where you can source open access or public domain maps:
* U.S. Geological Survey (USGS) - All maps are in the public domain. (*[*http://www.usgs.gov*](http://www.usgs.gov/)*)
* PlaniGlobe - All maps are published under a Creative Commons license so please cite u201cPlaniGlobe,* [*http://www.planiglobe.com*](http://www.planiglobe.com/)*, CC BY 2.0u201d in the image credit after the caption. (*[*http://www.planiglobe.com/?lang=enl*](http://www.planiglobe.com/?lang=enl)*)
* Natural Earth - All maps are public domain. (*[*http://www.naturalearthdata.com/about/terms-of-use/*](http://www.naturalearthdata.com/about/terms-of-use/)*).*

Basemap shapefile downloaded from GADM at <https://gadm.org> and GADM license allowing use is below (<https://gadm.org/license.html>):

“The data are freely available for academic use and other non-commercial use. Redistribution or commercial use is not allowed without prior permission.

Using the data to create maps for publishing of academic research articles is allowed. Thus you can use the maps you made with GADM data for figures in articles published by PLoS, Springer Nature, Elsevier, MDPI, etc. You are allowed (but not required) to publish these articles (and the maps they contain) under an open license such as CC-BY as is the case with PLoS journals and may be the case with other open access articles. Data for the following countries is covered by a a different license Austria: Creative Commons Attribution-ShareAlike 2.0 (source: Government of Austria)” *8) We note that you have indicated that there are restrictions to data sharing for this study. For studies involving human research participant data or other sensitive data, we encourage authors to share de-identified or anonymized data. However, when data cannot be publicly shared for ethical reasons, we allow authors to make their data sets available upon request. For information on unacceptable data access restrictions, please see*
[*https://journals.plos.org/plosntds/s/data-availability#loc-unacceptable-data-access-restrictions*](https://journals.plos.org/plosntds/s/data-availability#loc-unacceptable-data-access-restrictions) *Before we proceed with your manuscript, please address the following prompts:

 a) If there are ethical or legal restrictions on sharing a de-identified data set, please explain them in detail (e.g., data contain potentially identifying or sensitive patient information, data are owned by a third-party organization, etc.) and who has imposed them (e.g., a Research Ethics Committee or Institutional Review Board, etc.). Please also provide contact information for a data access committee, ethics committee, or other institutional body to which data requests may be sent.*

I have coordinated with our higher headquarters and received guidance allowing publication of a deidentified dataset per request. *b) If there are no restrictions, please upload the minimal anonymized data set necessary to replicate your study findings to a stable, public repository and provide us with the relevant URLs, DOIs, or accession numbers. Please see* [*http://www.bmj.com/content/340/bmj.c181.long*](http://www.bmj.com/content/340/bmj.c181.long) *for guidelines on how to de-identify and prepare clinical data for publication. For a list of recommended repositories, please see* [*https://journals.plos.org/plosone/s/recommended-repositories*](https://journals.plos.org/plosone/s/recommended-repositories)*. You also have the option of uploading the data as Supporting Information files, but we would recommend depositing data directly to a data repository if possible.

 Please update your Data Availability statement in the submission form accordingly.*

I have updated the Data Availability statement that data will be published to a public repository upon acceptance.

*9) Please amend your detailed Financial Disclosure statement. This is published with the article, therefore should be completed in full sentences and contain the exact wording you wish to be published.
i). State what role the funders took in the study. If the funders had no role in your study, please state: “The funders had no role in study design, data collection and analysis, decision to publish, or preparation of the manuscript.”
ii). If any authors received a salary from any of your funders, please state which authors and which funders.*

Have updated the Financial Disclosure as advised above and also included in the manuscript that ““The funders had no role in study design, data collection and analysis, decision to publish, or preparation of the manuscript.”
