# Supplementary material for: Continuous detection of Chikungunya Virus in a passive surveillance system in southern Thailand, 2012–2019
Source: PLoS Negl Trop Dis. 2025 Jan 7;19(1):e0012776. doi: 10.1371/journal.pntd.0012776 (PMC11741575; doi:10.1371/journal.pntd.0012776)
Supplement: Attachment — Submitted filename: Response to Reviewers 14 Sep 2024.docx [file pntd.0012776.s006.docx]

Response to Reviewers: PLOS Neglected Tropical Diseases submission (PNTD-D-24-00295R1)
Corresponding Author: Aaron Farmer
Date: 14 September 2024

Dear Editors,

Thank you for the opportunity to review and responded to your inquiries. Please see the responses below and we look forward to additional questions or concerns.

Sincerely

Aaron Farmer, DO MPH FIDSA

Review and Response:

*Reviewer #1: It would be helpful if authors prepare a diagram to show the flow of diagnostic assay to make it easier for readers to understand the methods of this surveillance study*

Thank you for your suggestion. Figure 2 added to the manuscript.


*Reviewer #1: 1. It is quite surprising that there is no difference in the frequency of myalgia and arthralgia in CHIK and non-CHIK groups. Also, it makes sense that the frequency during epidemic seasons were higher than interepidemic (although only for myalgia, as the p value in arthralgia is 0.045). I think you should mention the tendency of arthralgia to be more frequent in the epidemic group, although not significant. Readers do not have to go to the supplement to find out the p value. In my opinion it is important to discuss these findings (myalgia/arthralgia in CHIK/non CHIK and in epidemic/inter epidemic, as these clinical findings are known to be the prominent symptoms of CHIKV infections.*

Thank you for this question. We performed a reanalysis of the data and on review, identified that the rate of myalgia was significantly different with rates of 32.3 vs 23.2% (p<0.01). To simplify the reading, we have added this information to Table 2 and the manuscript and removed Supplemental Figure 2. Even after re-analysis, arthralgia rates were still low and we have discussed the low rates briefly in the “Discussion” section. We hesitate to draw any conclusions given the design of the study and potential limitations of passive surveillance and as mentioned by the reviewer, these findings are interesting and would be better defined through prospective, longitudinal studies.

*2. Authors have discussed why you chose HI instead of PRNT for diagnosing (more practical). It will be informative for readers if you also add why you did not choose ELISA method, which is even more practical than HI, and has been widely used?*

Although ELISA is indeed practical for quantifying IgM and IgG antibody levels, as it provides data on the presence and concentration of antibodies, it does not assess the functionality of these antibodies. In contrast, HAI evaluates the functional ability of antibodies to inhibit CHIKV-induced hemagglutination, which is crucial for understanding their protective potential. Furthermore, the strong correlation between HAI and PRNT assays means that HAI can offer valuable insights into the functional efficacy of antibodies, making it a more informative choice for assessing protective immunity against CHIKV compared to ELISA.

Added to Lines 158-159: HAI was chosen over ELISA to allow assessment of functional antibody response to CHIKV.

*3. It is important for authors to discuss why there were many specimens during acute illness that were missed by RT-PCR (average negative by RT-PCR was 2.6 days after onset of illness), suggesting that there were quite many specimens obtained before 2.6 days negative by RT-PCR.*

It is unclear the cause of the negative RT-PCR given the lab was obtained shortly after symptom onset. We have noted some considerations for the low rate of viral RNA capture in the limitations section and it is also possible that the sample was degraded in transit or during storage as some samples were noted to have hemolysis which could negatively affect molecular testing. The detection of other samples during the same time frame that were positive argues against widespread laboratory errors and quality control performed at the time of testing did not note any deficiencies. We have added to the limitations section to reflect our thoughts on reasons for the discrepancy.

*4. Several inconsistencies between results and method: e.g. a) in the method section, the max day of convalescent specimens is 21 days apart, but in the result section is 35 days (line 204). b) in the method, PRNT was conducted in acute specimens randomly chosen, but you compared the results of HI and PRNT of the convalescent specimens. c) line 141-142: HI was performed when acute and convalescent specimens are available, but the previous sentence stated when convalescent specimens are not available, they are not included in the study. d) line 148-150, and line 176-177 PCR was conducted in acute and convalescent specimens?*

a) The max day in the results was corrected to 21 days due to a typographical error.
b) Line 159 (updated to Line 161-162 in current version) should read “acute febrile illness” rather than just “acute.” These were acute and convalescent samples obtained from acute febrile illness participants. Results were compared in convalescent samples to assess changes in titer.
c) Removed the fragment “if paired acute and convalescent samples were available” to clarify that any samples meeting criteria with sufficient volume were tested with serological methods.
d) PCR is from acute specimens only.

*5. In the response to the reviewers, authors explain that cases confirmed by RT-PCR only was 90 (6.1%). Does it mean, HI results were not consistent with RT-PCR results, or they were not tested for HI?. Authors also did not response to the question from reviewers regarding the discrepancies of PRNT and HI. Instead, authors explained that the correlation was 0.817. Does it suggest that they are some discrepancies? When it occurred (e.g. HI positive, but PRNT negative), what the diagnostic decision was?*

We apologize for the prior oversight. Yes, there were some discrepancies between the HI, PRNT and PCR results as expected for laboratory assays. For HAI testing, acute and convalescent samples may detect seroconversion in samples that were not detectable by a single acute RT-PCR. There was also some discrepancy between HAI and PRNT even though they had a strong correlation, identified as 0.817. In situations where there were discrepancies between HAI and RT-PCR, RT-PCR was used; for HAI vs PRNT, PRNT was used as it is the more specific test. As each assay has it’s own limitations, we used a combination to obtain the greatest level of sensitivity and specificity.

*6. Regarding the proportion of dengue cases (17.5%), it is not clear the denominator that is used. Authors say from all cases (>2127 or 1473 subjects). Without the denominator, it is hard to understand this sentence:" Acute DENV infection was confirmed by RT-PCR in 258 (17.5%) of all participants presenting with acute febrile illness. Among these, 3 were also RT-PCR-positive for CHIKV, for a PCR-confirmed coinfection rate of 1.2%"
Was dengue confirmed by RT-PCR only, or also serological assay?. Can we compare the results from dengue and chikungunya cases?*

The denominator used in this was 1473 which was total participants meeting study inclusion criteria. Dengue was tested using both RT-PCR and serology assays but further discussion was limited as the focus of the manuscript was on the prevalence of chikungunya infection. In general, the dengue data was included to provide support for the ongoing arboviral disease in the area and validity of the overall sample collection process. We do intend to provide the dataset used for this study if additional analysis is needed by others.

*7. It is hard to understand this sentence:
In the text: Among the 1473 individuals that presented with febrile illness from 2012-2019 and were eligible for inclusion in this analysis, 401 (27.3%) had confirmed acute CHIKV infection by 202 molecular and/or serological methods (PCR and/or acute HAI sample positive); of these 90 of 1473 (6.1%) of individuals had detectable CHIKV RNA in acute specimens by RT-PCR.
In the response to reviewers:*

We have reviewed the data (which will be provided to data repository upon acceptance) and revised the numbers as per below. We have updated the text (Lines 226-228 in the clean version) and also included these numbers in Figure 2 for ease of understanding.

a) PCR AND Serology- 45/1466 (3.1%)
b) PCR only- 45/1466 (3.1%)
c) Serology only- 308/1466 (21.0%)

*8. As I already suggest in the method section, it will be easier if authors provide a figure that explains the flow of the tests and the results as well.*

This has been added as Figure 2.
